# Supplementary material for: Selection for toxin production in spatially structured environments increases with growth rate
Source: ISME J. 2025 Apr 8;19(1):wraf061. doi: 10.1093/ismejo/wraf061 (PMC12041421; doi:10.1093/ismejo/wraf061)
Supplement: supplemental-materials_wraf061 [file supplemental-materials_wraf061.pdf]

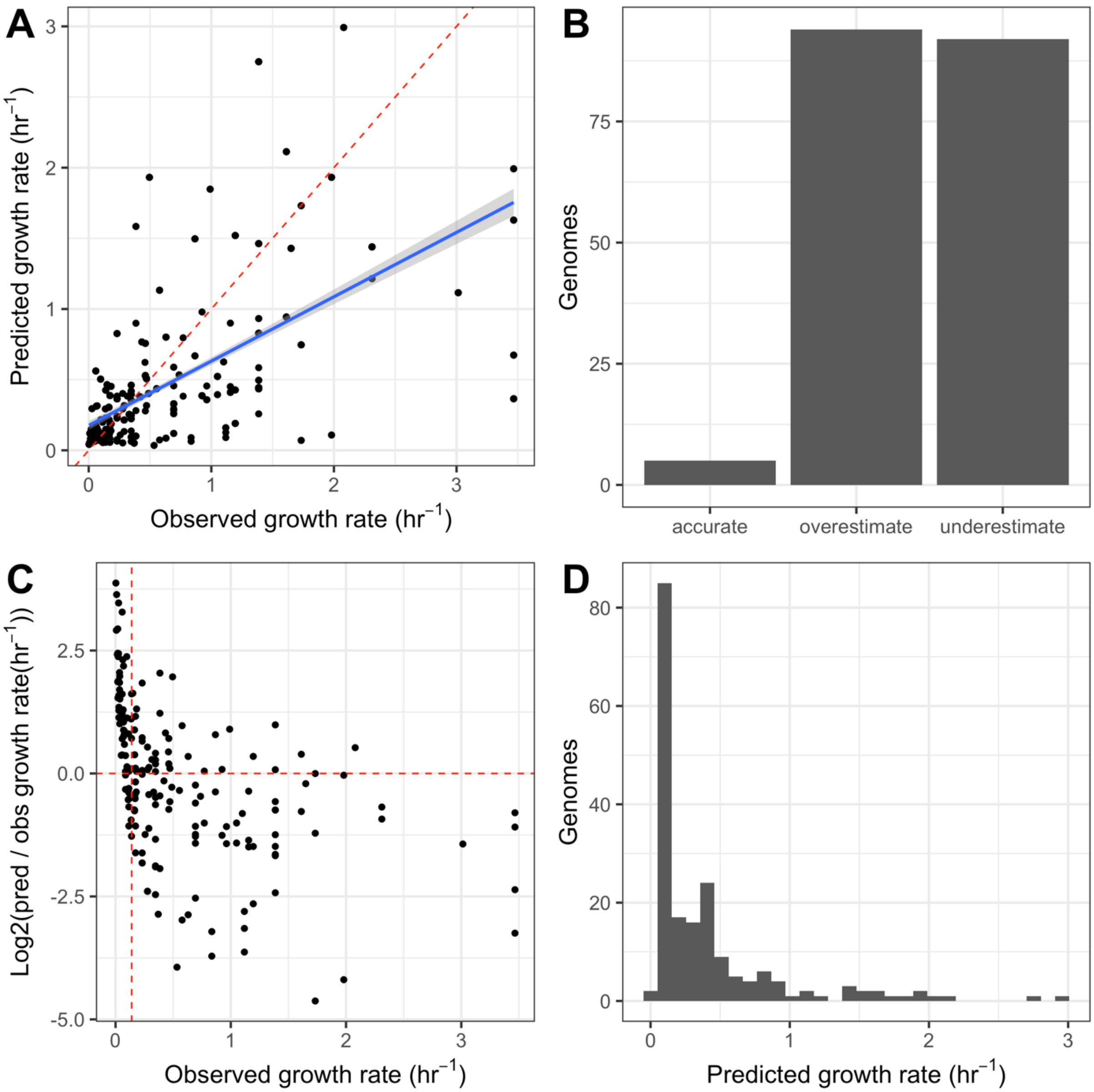


**Supplemental Fig. S1. The Vieira-Silva and Rocha dataset demonstrates the predictive limitation of gRodon. A:** Scatterplot of gRodon predictions of growth rate (hr$^{-1}$) versus observed growth rates (hr$^{-1}$) for the Vieira-Silva and Rocha dataset. The dashed red line represents perfect predictions, while the blue line corresponds to the actual linear relationship between values. **B:** When compared to experimental values, gRodon perfectly predicted (within 2 significant figures) the growth rate of 5 species, overestimated the growth rate of 94 species, and underestimated the growth rate of 92 species. **C:** Log2-foldchange in predicted growth rate (hr$^{-1}$)

compared to observed growth rate ($hr^{-1}$). Values above the horizontal red line indicate overpredictions by gRodon, while values below the red line indicate underestimates. gRodon is reported to underestimate actual maximum doubling times, with predictions being most accurate when doubling times are less than 5 hours (or, organisms grow faster than a rate of 0.14 $hr^{-1}$, points right of the vertical red line). Based on the Vieira-Silva and Rocha dataset, when observed growth rate is slow (< 0.14 $hr^{-1}$), gRodon tends to overestimate growth rate. As observed growth rate increases, gRodon instead underestimates growth rate. **D:** Histogram of gRodon-predicted growth rates for the Vieira-Silva and Rocha dataset (mean 0.41, median 0.21, min 0.035, max 3.0). Predicted growth rates did not capture either the maximum or minimum values of observed growth rates. We therefore expected that the use of gRodon-predicted rates for the *Streptomyces* dataset should somewhat overestimate the probability of the presence or absence of a given biosynthetic gene cluster by truncating the true variance in growth rate.

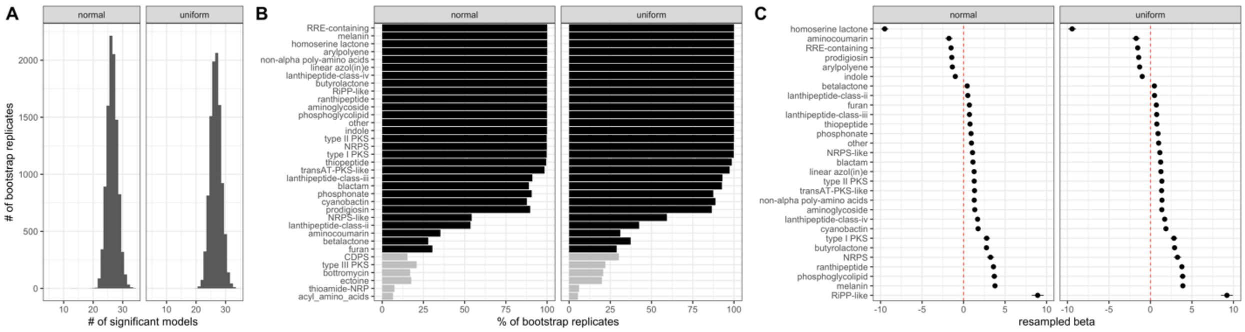


**Supplemental Fig. S2. In the *Streptomyces* dataset, 19 classes of BGCs are robustly correlated with growth rate following two different bootstrapping approaches. A:** Histogram of total number of statistically significant models across 10,000 bootstrapping replicates for two resampling approaches. Using a uniform distribution for resampling, the median number of significant models per replicate is 27 (mean = 26.7, maximum = 34, minimum = 20). Using a normal distribution for resampling, the median number of significant models per replicate is 26 (mean = 26.5, maximum = 34, minimum = 5). **B:** Percent of times the presence of a BGC class is significantly correlated with growth rate across 10,000 bootstrapping replicates for two resampling approaches. Only BGC classes that were significant in >5% of bootstrapping replicates for at least one resampling approach are shown. BGC classes that were significantly correlated with growth rate using our initial dataset without resampling are colored in black (**Fig. 5B**), and classes that were not significantly correlated with growth rate in our initial dataset without resampling are colored in gray. The 19 classes of BGCs that are significantly correlated with growth rate in at least 95% of bootstrapping replicates for both resampling approaches are considered truly significant (**Fig. 5B**). **C**: The distribution of $\beta$ coefficients for logistic regression models generated for growth rate and BGC class presence following two resampling approaches. Points represent the mean estimate across all 10,000 bootstrapping replicates and line segments indicate the standard deviation across replicates. Only the 29 BGC classes that were initially significantly correlated with growth rate (**Fig. 5B**) are shown.

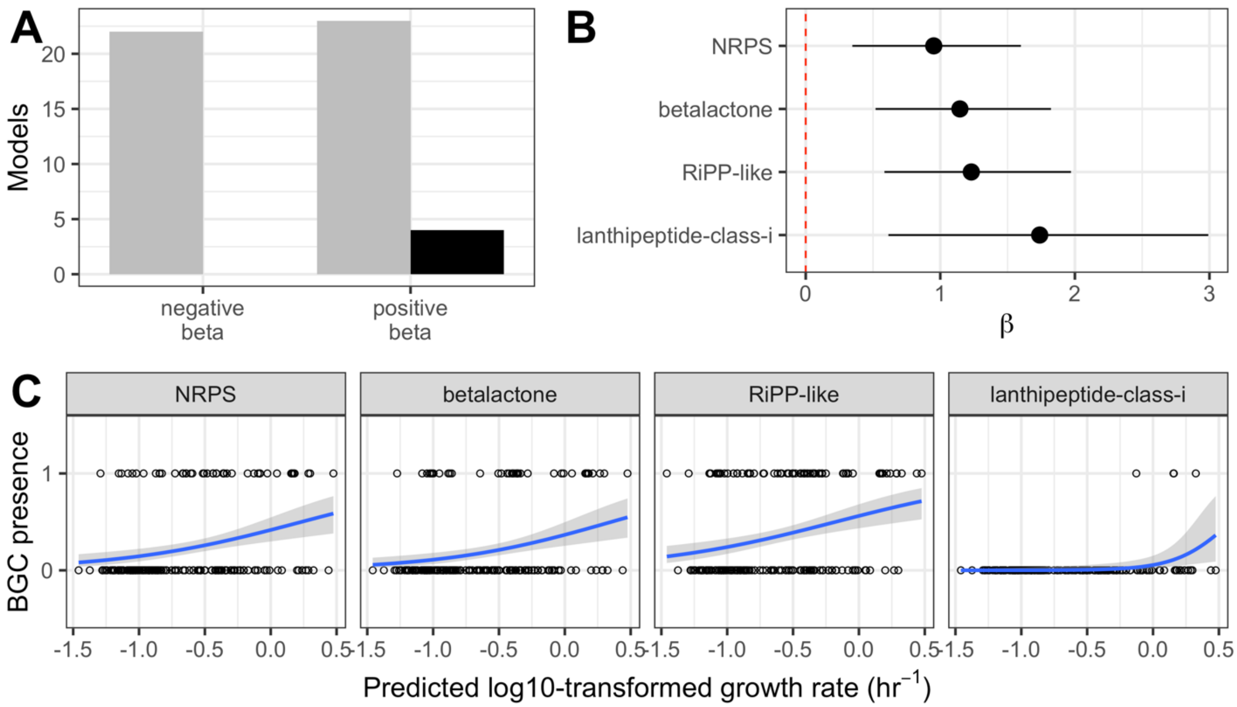


**Supplemental Fig. S3. In the Vieira-Silva and Rocha dataset, three of five biosynthetic gene clusters are also predicted to be correlated with growth rate when gRodon-predicted growth rates are used for model generation. A:** Number of logistic regression models with negative and positive $\beta$ values when gRodon-predicted maximum growth rate values are used in place of observed growth rates. The number of models with significant correlations (following Benjamini-Hochberg correction) are colored in black. **B:** $\beta$ coefficients for logistic regression models generated for growth rate and BGC class presence. Line segments indicate 95% confidence intervals for model estimates. All BGC classes can potentially encode toxins. Three of them were also significantly correlated wth growth rate when observed maximum growth rate was used - betalactones, NRPS, and lanthipeptide class I BGCs – while RiPP-like clusters were not (**Fig. 4B**). Ectoines and siderophores were significantly correlated using observed growth rate but not predicted growth rate. The use of predicted growth rates also increased the value of $\beta$ coefficients for the BGC classes that were significant in both cases, underscoring the differences

in both the underlying distribution of the data and the resulting probability distribution. **C:** Regression plots of the four BGC classes with a significant correlation, in order of increasing $\beta$ coefficient. Predicted growth rates are log10-transformed to better observe the range of small values because growth rates across the dataset are left-skewed.

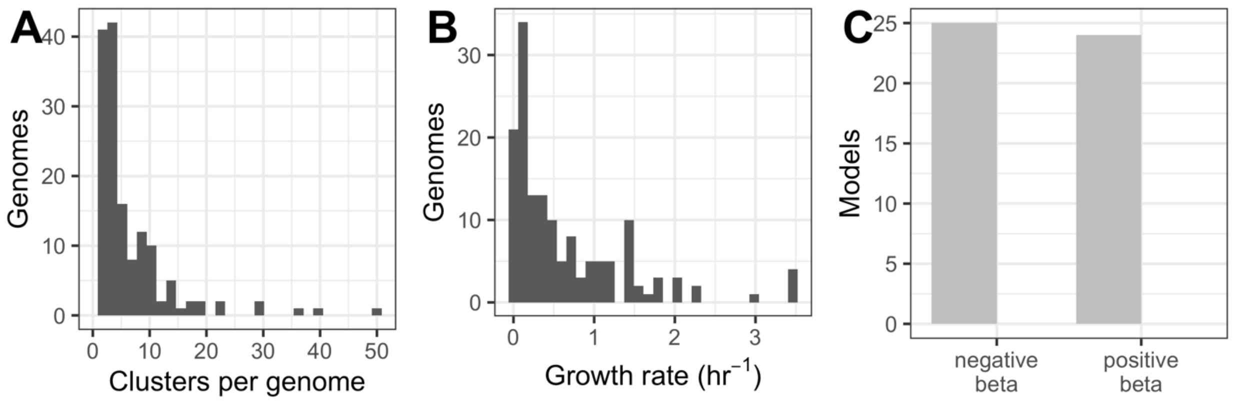


**Supplemental Fig. S4. In the Vieira-Silva and Rocha dataset, removing genomes with no detected biosynthetic gene clusters reduces the power of the analysis and eliminates significant correlations between growth rate and presence/absence of certain BGC classes.** **A:** Histogram of total number of detected BGC classes per genome, excluding genomes with no detected BGCs. When genomes without predicted BGCs are excluded, only 148 unique genomes remain in the dataset. The minimum number of BGCs detected in a genome was 1, with a maximum of 50, a mean of 6.5, and a median of 4. **B:** Histogram of growth rates for each dataset, excluding genomes with no detected BGCs. In this reduced dataset, observed growth rates ranged from 0.003 to 3.47 hr$^{-1}$, with a median of 0.35 and a mean of 0.67. **C:** Number of logistic regression models with negative and positive $\beta$ values. The number of models with significant correlations (following Benjamini-Hochberg correction) are colored in black, though there are none due to the reduced power of the analysis.

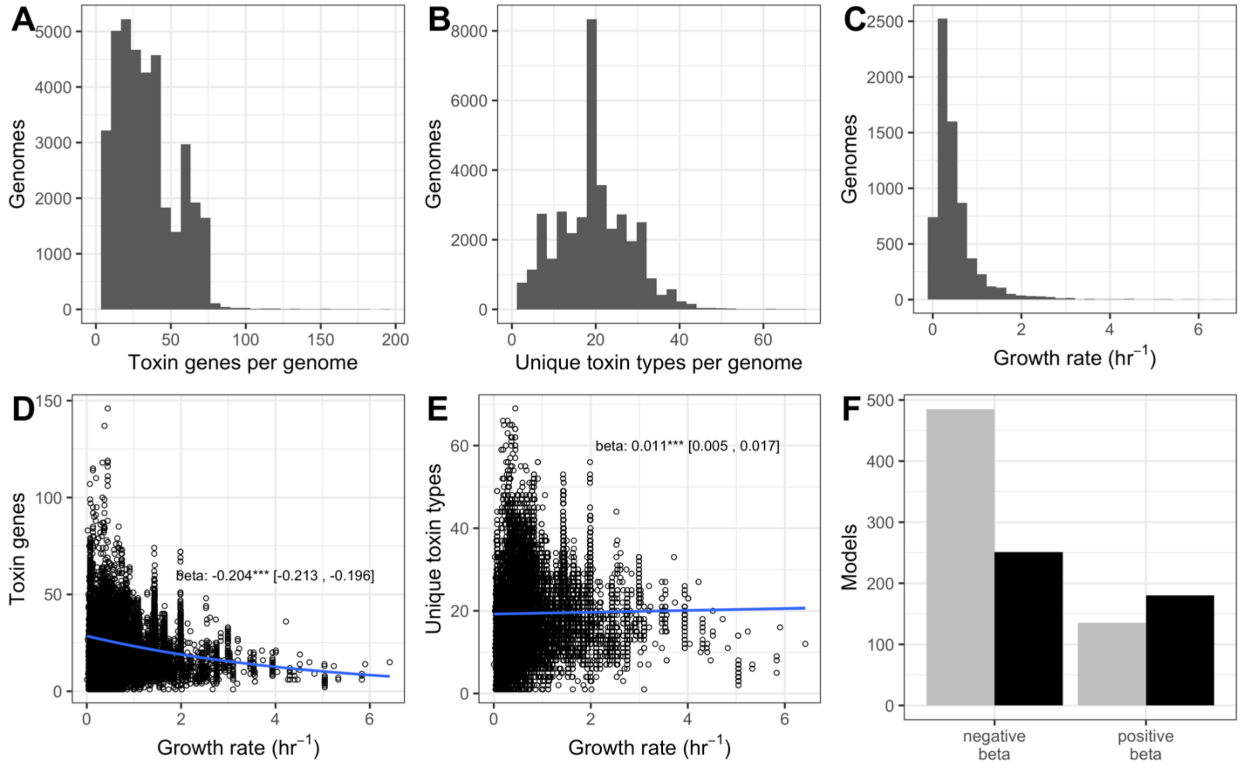


**Supplemental Fig. S5. In a large dataset of predicted toxin genes, the number of unique types of toxin genes present in a genome is weakly correlated with growth rate. A:** Histogram of total number of predicted toxin genes per genome (minimum of 1, maximum of 193, mean of 32.88, median of 29). **B:** Histogram of number of unique types of predicted toxin genes per genome (minimum of 1, maximum of 69, mean of 19.37, median of 19). **C:** Histogram of predicted growth rates across all species represented (minimum of 0.0032, maximum of 6.42, mean of 0.496, median of 0.348). **D:** Generalized linear regression model (poisson family) between growth rate and number of toxin genes present in a genome, with an estimate of -0.204 and a 95% confidence interval of [-0.213, -0.196]. 95% confidence intervals were based on 1,000 bootstrapping replicates. **E:** Generalized linear regression model (poisson family) between growth rate and number of unique types of toxin genes present in a genome, with an estimate of 0.011 and a 95% confidence interval of [0.005, 0.0017]. 95% confidence intervals were based on

1,000 bootstrapping replicates. **F:** Number of logistic regression models (binomial family) with negative and positive $\beta$ values. The number of models with significant correlations (following Benjamini-Hochberg correction) are colored in black. The presence of proteins with a given Pfam ID were used for model generation.

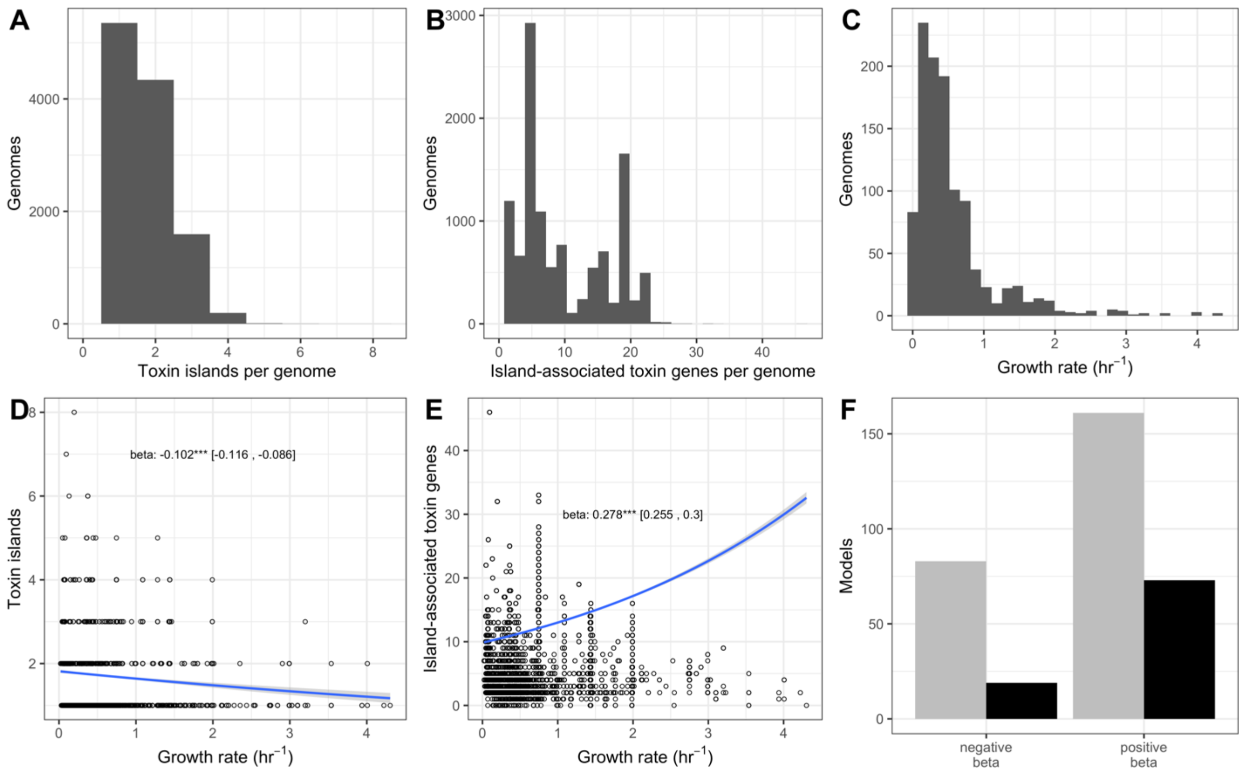


**Supplemental Fig. S6. In a large dataset of predicted toxin islands, the number of island-associated toxin genes present in a genome is weakly correlated with growth rate. A:** Histogram of total number of predicted toxin islands per genome (minimum of 1, maximum of 8, mean of 1.71, median of 2). **B:** Histogram of number of island-associated toxin genes per genome (minimum of 0, maximum of 46, mean of 9.68, median of 7). **C:** Histogram of predicted growth rates across all species represented (minimum of 0.02, maximum 4.3, mean of 0.55, median of 0.38). **D:** Generalized linear regression model (poisson family) between growth rate and number of toxin islands present in a genome, with an estimate of -0.101 and a 95% confidence interval of [-0.116, -0.086]. 95% confidence intervals were based on 1,000 bootstrapping replicates. **E:** Generalized linear regression model (poisson family) between growth rate and number of unique types of toxin genes present in a genome, with an estimate of 0.278 and a 95% confidence interval of [0.255, 0.3]. 95% confidence intervals were based on

1,000 bootstrapping replicates. **F:** Number of logistic regression models (binomial family) with negative and positive $\beta$ values. The number of models with significant correlations (following Benjamini-Hochberg correction) are colored in black. Toxin island presence was used for model generation.

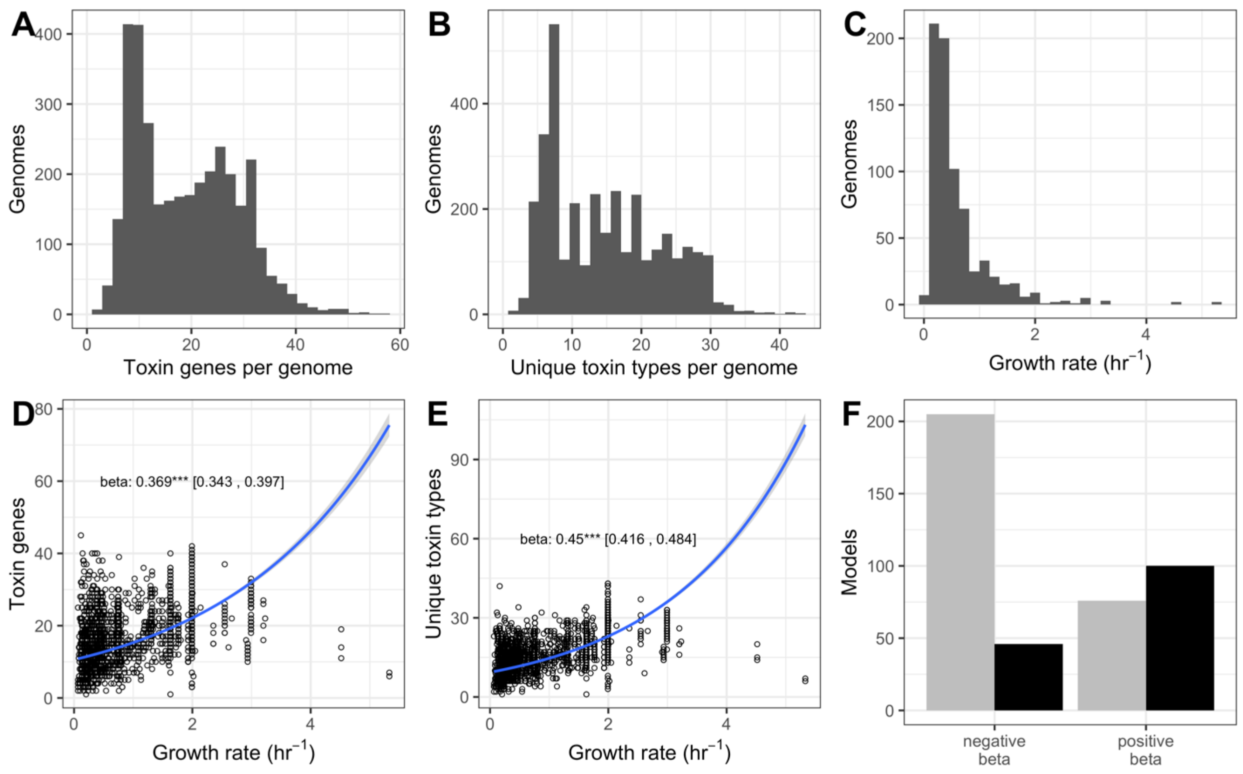


**Supplemental Fig. S7. In a large dataset of marine taxa, both the number of toxin genes and unique types of toxin genes in a genome are weakly positively correlated with growth rate. A:** Histogram of total number of predicted toxin genes per genome (minimum of 1, maximum of 57, mean of 18.77, median of 18). **B:** Histogram of number of unique types of predicted toxin genes per genome (minimum of 1, maximum of 43, mean of 14.81, median of 14). **C:** Histogram of predicted growth rates across all species represented (minimum of 0.066, maximum of 5.33, mean of 0.599, median of 0.41). **D:** Generalized linear regression model (poisson family) between growth rate and number of toxin genes present in a genome, with an estimate of 0.369 and a 95% confidence interval of [0.343, 0.397]. 95% confidence intervals were based on 1,000 bootstrapping replicates. **E:** Generalized linear regression model (poisson family) between growth rate and number of unique types of toxin genes present in a genome, with an estimate of 0.45 and a 95% confidence interval of [0.416, 0.484]. 95% confidence

intervals were based on 1,000 bootstrapping replicates. **F:** Number of logistic regression models (binomial family) with negative and positive $\beta$ values. The number of models with significant correlations (following Benjamini-Hochberg correction) are colored in black. The presence of proteins with a given Pfam ID were used for model generation.

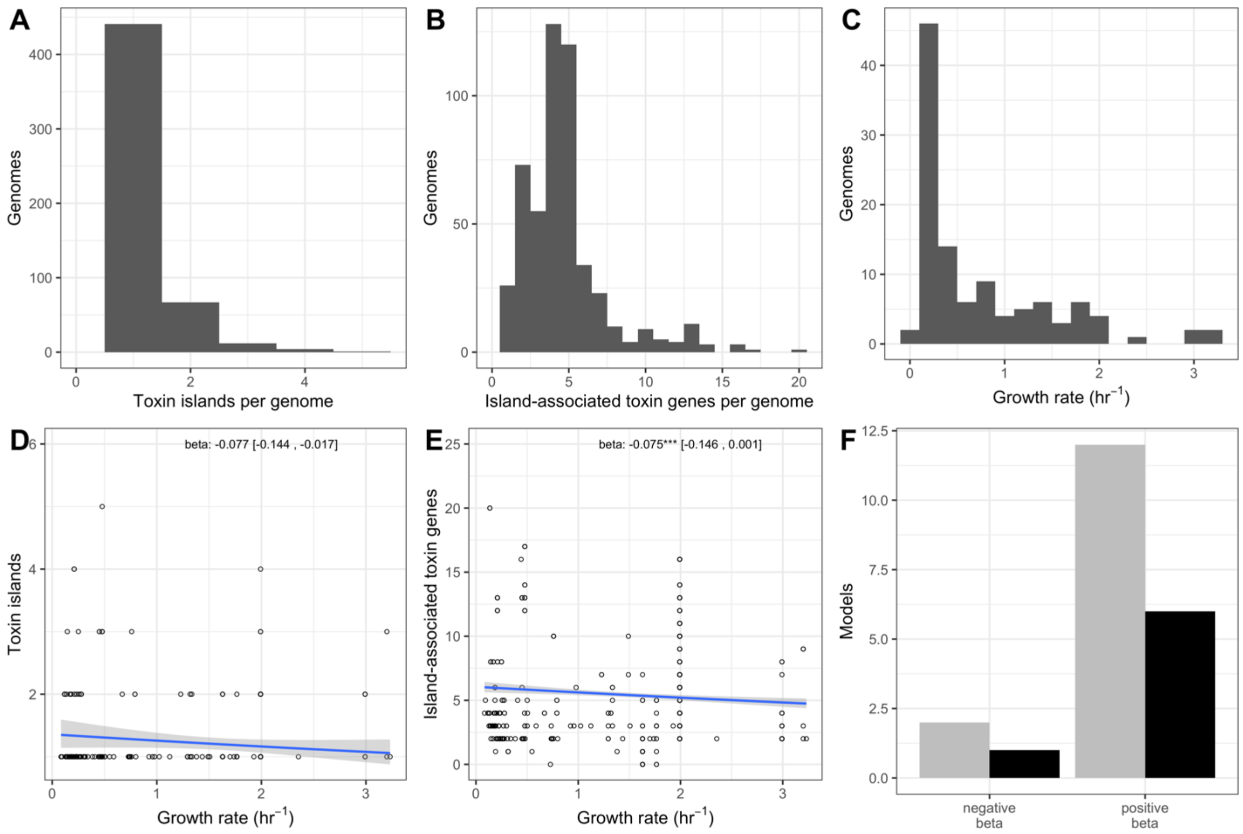


**Supplemental Fig. S8. In a large dataset of marine taxa, the number of toxin islands and the number of island-associated toxin genes in a genome are both negatively correlated with growth rate. A:** Histogram of total number of predicted toxin islands per genome (minimum of 1, maximum of 5, mean of 1.2, median of 1). **B:** Histogram of number of island-associated toxin genes per genome (minimum of 0, maximum of 20, mean of 4.56, median of 4). **C:** Histogram of predicted growth rates across all species represented (minimum of 0.084, maximum of 3.23, mean of 0.74, median of 0.45). **D:** Generalized linear regression model (poisson family) between growth rate and number of toxin genes present in a genome, with a non-significant estimate of -0.077 and a 95% confidence interval of [-0.144, -0.017]. 95% confidence intervals were based on 1,000 bootstrapping replicates. **E:** Generalized linear regression model (poisson family) between growth rate and number of unique types of toxin genes present in a genome, with an estimate of -0.075 and a 95% confidence interval of [-0.146,

-0.001]. 95% confidence intervals were based on 1,000 bootstrapping replicates. **F:** Number of logistic regression models (binomial family) with negative and positive $\beta$ values. The number of models with significant correlations (following Benjamini-Hochberg correction) are colored in black. Toxin island presence was used for model generation.

**SUPPLEMENTAL METHODS**

To increase the breadth of our comparative genomics analysis, we evaluated the dataset presented in the 2023 Danov et al. paper "Toxinome–the bacterial protein toxin" [18]. This paper assembled a database of 175,573 toxin proteins from a variety of protein databases. Proteins were clustered into ~70,000 groups and mapped to genomes in the Integrated Microbial Genomic database [19], resulting in a final database of >1,000,000 toxins identified across 59,475 genomes representing ~8,000 microbial species. To leverage this dataset, we used the ncbi_datasets command line tool (ncbi_datasets v. 16.24.1) in August 2024 to download reference genomes for as many species as possible represented in the database. We downloaded reference genomes (fna files) for all species for which an annotated reference genome was available on GenBank, leaving us with a dataset of 5,741 species represented by 37,028 unique genomes. GenBank fna files were used as input into gRodon v. 2.0.0 [17]. We aggregated a final dataset by associating individual bacterial strains with the predicted growth rate of its reference genome. For example, 5,014 *Staphylococcus aureus* genomes are represented in the Toxinome dataset, each with a different suite of putative toxin genes. For each of those 5,014 genomes, we assumed that the gRodon-predicted doubling time of the NCBI *S. aureus* reference genome provided a reasonable estimation of growth rate for all individual strains. Lastly, we cleaned the Toxinome dataset to limit it to toxin genes that should be most likely to be used in interference competition by removing products that appeared to correspond solely to plasmid maintenance. Our final dataset consisted of IMG genome IDs, species names, reference-genome gRodon-predicted growth rates, Pfam IDs, unique protein IDs, and toxin gene product names. We also considered the Toxinome dataset outlining predicted toxin islands, which were identified in 11,496 of the 37,028 genomes for which we had derived gRodon-predicted growth rates.

To investigate whether there was a correlation between growth rate and toxin maintenance, we calculated the total number of toxin genes (unique protein IDs) identified per genome, as well as the total number of unique types of toxin genes per genome (unique Pfam IDs) (**Supplemental Fig. S5A-C**). Generating generalized linear regression models (poisson family, appropriate for counts data) using these data, we found that there was a weak negative correlation between the number of toxin genes in a genome and growth rate and a weak positive correlation between the number of unique types of toxin genes in a genome and growth rate (**Supplemental Fig. S5D-E**). These results were robust to resampling following 1,000 bootstrapping replicates (**Supplemental Fig. S5D-E**). We expected that the number of unique types of toxin genes may be a better overall representation of selection for toxin maintenance, indicating the diversity of interference competition systems necessary for a genome's success, while the total number of individual toxin genes could be more likely to be confounded by species- or strain-specific rates of genomic evolution. We also noted that the poor fit between growth rate and toxin gene presence was not surprising given the significant oversampling of the data, which is enriched for slow-growing genomes. Finally, we recapitulated our logistic regression analysis, using Pfam IDs in place of BGCs to generate logistic regression models (binomial family, link logit) between predicted growth rates and the presence of a protein with a given Pfam ID in a genome. We calculated the number of significant models with negative and positive $\beta$ values following Benjamini-Hochberg p-value correction for multiple comparison (**Supplemental Fig. S5F**). This analysis suggested that the presence of most types of toxin genes were negatively correlated with growth rate, contrary to our modeling expectations.

One potential limitation of the Toxinome dataset is that it is not structured to easily group Pfam IDs by relevance to produce a given toxin. However, the database also includes a dataset of

predicted toxin islands, regions of the genome where toxin/antitoxin genes cluster. We expected that toxin islands could be more representative of the suite of genes required to produce a given toxin and therefore a more appropriate proxy for a toxin production phenotype. For that reason, we also evaluated the relationship between predicted toxin islands and growth rate in the Toxinome dataset. As we have noted, toxin islands were not predicted in every genome, reducing the size of the dataset. The Toxinome database identified ~3,200 unique predicted toxin islands across a subset of 11,496 genomes for which we had gRodon-predicted growth rates. We calculated the total number of toxin islands per genome, as well as the total number of toxins per genome associated with a toxin island (**Supplemental Fig. S6A-C**). We generated generalized linear regression models (poisson family, appropriate for counts data) using these data and found that there was a weak negative correlation between the number of toxin islands in a genome and growth rate and a weak positive correlation between the number of island-associated toxin genes in a genome and growth rate (**Supplemental Fig. S6D-E**). These results were robust to resampling following 1,000 bootstrapping replicates (**Supplemental Fig. S5D-E**). We again recapitulated our logistic regression analysis, using toxin islands in place of BGCs to generate logistic regression models (binomial family, link logit) between predicted growth rates and the presence of toxin island in a genome. We calculated the number of significant models with negative and positive $\beta$ values following Benjamini-Hochberg p-value correction for multiple comparison (**Supplemental Fig. S6F**). In this case, the presence of many toxin islands were significantly positively correlated with growth rate.

Finally, we repeated the previously described statistical tests on a subset of the Toxinome dataset. Any species in the full Toxinome dataset belonging to the orders *Flavobacteriales, Oceanospirillales, Campylobacterales, Vibrionales*, and *Alteromonadales*, or to the classes

Alphaproteobacteria or Actinobacteridae, were included in the subset (a full list of taxon names is available in **Supplemental Table S2**). These orders and classes were chosen because they are found predominantly in marine environments and are often particularly abundant in marine snow and marine sediments [22-27, 56-57]. The final dataset consisted of 3,427 genomes representing 639 species (**Supplemental Fig. S7A-C**). In this subset, we found that both the total number and number of unique types of toxin genes were weakly positively correlated with growth rate (**Supplemental Fig. S7D-E**) and that these correlations were robust to bootstrap resampling (1,000 replicates). However, the total number of toxin islands and toxin-associated genes were both negatively correlated with growth rate (**Supplemental Fig. S8D-E**). We also observed that the presence of a majority of Pfam IDs and toxin islands were positively correlated with growth rate, in alignment with our modeling expectations and in contrast to our findings with the full Toxinome dataset (**Supplemental Fig. S7F, S8F**). Altogether, these results suggested that the relationship between growth rate and toxin production is likely very weak, although this analysis was still broadly supportive of our modeling results. Additionally, our analysis suggested that the presence of many toxin islands is positively correlated with growth rate in both the full dataset and the marine taxa subset, a metric we expected to be analogous to the BGC identification we used as a proxy for toxin production in our main datasets.
